# Evaluation of global health capacity building initiatives in low-and middle-income countries: A systematic review

**DOI:** 10.7189/jogh.10.020412

**Published:** 2020-12

**Authors:** Hady Naal, Maria El Koussa, Melissa El Hamouch, Layal Hneiny, Shadi Saleh

**Affiliations:** 1 Global Health Institute at the American University of Beirut, Beirut, Lebanon; 2 Saab Medical Library at the American University of Beirut, Beirut, Lebanon; 3 Faculty of Health Sciences at the American University of Beirut, Beirut, Lebanon

## Abstract

**Background:**

Low- and middle-income countries (LMICs) are in dire need to improve their health outcomes. Although Global Health Capacity Building (GHCB) initiatives are recommended approaches, they risk being ineffective in the absence of standardized evaluation methods. This study systematically reviews evaluation approaches for GHCB initiatives in LMICs.

**Methods:**

We searched the Medline (OVID), PubMed, Scopus, and Embase.com databases for studies reporting evaluation of a GHCB initiative in a LMIC from January 1, 2009 until August 15, 2019. To differentiate them from intervention, prevention, and awareness initiatives, included articles reported at least one approach to evaluate their learning modality. We excluded cross-sectional studies, reviews, and book chapters that only assessed the effect of interventions. Data identifying the learning modality, and evaluation method, level, time interval, and approach were extracted from articles as primary outcomes.

**Results:**

Of 8324 identified studies, 63 articles were eligible for analysis. Most studies stemmed from Africa and Asia (69.8%), were delivered and evaluated face-to-face (74.6% and 76.2%), mainly to professionals (57.1%) and community workers (20.6%). Although the use of online and blended modalities showed an increase over the past 4 years, only face-to-face initiatives were evaluated long-term beyond individual-level. GHCB evaluations in general lacked standardization especially regarding the tools.

**Conclusion:**

This is an important resource for evaluating GHCB initiatives in LMICs. It synthesizes evaluation approaches, offers recommendations for improvement, and calls for the standardization of evaluations, especially for long-term and wider impact assessment of online and blended modalities.

Low- and middle-income countries (LMICs) face increasing health challenges, coupled with a reduced ability to manage them [1,2]. According to the World Health Organization (WHO), there are around 57 LMICs experiencing significant health care crises and shortages of their health workforce [3,4]. LMICs are in dire need of a competent health workforce that has the capability to overcome the shortcomings of their fragile health care systems [1,2]. While global health is a multidisciplinary field that aims to resolve transnational health challenges for people worldwide, the health workforce refers to decision-makers and service providers who engage in action to enhance the health of populations [5,6]. The means to implement Global Health Capacity Building (GHCB) initiatives are becoming increasingly available given the ubiquitous advancements in technology and delivery methods [7,8]. GHCB initiatives are activities directed towards developing the abilities of individuals, organizations, and communities to manage global health-related issues [9,10]. These efforts are among the most important approaches to respond to such challenges in LMICs [11] since they have a large potential in improving health-related knowledge and skills, policies and practices, ultimately leading to changes in health care systems and health outcomes [11,12]. To develop, implement, and sustain these efforts, the use of adequate evaluation methods is necessary to gauge the effectiveness and impact of GHCB initiatives. However, little accounts of approaches used to evaluate GHCB initiatives in LMICs have been reported.

Current approaches to evaluate the impact of GHCB initiatives at the individual, organizational, and community levels have not been standardized. This is problematic because GHCB initiatives risk being ineffective and stagnant in the absence of adequate evaluation methods. This may also limit researchers’ and stakeholders’ ability to develop and upscale their own capacity building initiatives. Adequate evaluation is therefore a central component of GHCB initiatives and is an integral step to examine the impact associated with them [12]. In light of the aforementioned shortcomings, the present study systematically reviews and critically appraises the literature in order to identify and summarize commonly used evaluation methods for GHCB initiatives.

## METHODS

### Search strategy and selection criteria

This systematic review conforms to the Preferred Reporting Items for Systematic reviews and Meta-Analysis (PRISMA) [13]. The search strategy was conducted by a medical librarian (LH) to identify relevant studies using the following databases: Medline (OVID), Embase.com, PubMed, and Scopus. The search was restricted by date only, and included articles published between January 1, 2009 until the day of the final search on August 19, 2019. According to the Best Evidence Medical Education (BEME) collaboration, a 10-year cut off is usually recommended for search strategies in systematic reviews [14]. The search strategy aimed to identify articles that addressed outcome evaluation methods used in GHCB initiatives in LMICs. Accordingly, Medical Subject Headings (MeSH) and keywords of the four concepts in our topic were combined in the search strategy (see Appendix S1 in the **Online Supplementary Document**), namely “Capacity Building”, “Global Health”, “Evaluation”, and “Low- and Middle-Income Countries”. We also searched Open Grey database for grey literature using a similar search strategy.

Studies were included if they were peer-reviewed journal articles, published in English. Articles were required to be related to GHCB, and specifically to report on outcome evaluation methods targeting at least one or more components of the GHCB learning modality (**Table 1**). Articles were excluded if they were editorials, commentaries, presentations, needs assessment or cross-sectional studies, prevention and intervention studies that did not evaluate the modality, poster abstracts, or books. Articles were also excluded if they were published before 2009, if they did not address an LMIC, if they were not in English, if they did not relate to GHCB, and if they did not report an outcome evaluation method. Finally, process evaluations, feasibility studies, and interventions that only reported intervention outcomes were excluded. While pre-and-post changes give insight into the effectiveness of the intervention, they do not tap into the learners’ reactions to and experiences with the GHCB initiative, which is this reviews’ primary focus.

**Table 1:** Definitions of key terms

Key terms	Definitions
Global Health	“Health problems, issues, and concerns that transcend national boundaries, which may be influenced by circumstances or experiences in other countries, and which are best addressed by cooperative actions and solutions” [15]
Capacity Building	“The development of knowledge, skills, commitment, structures, systems, and leadership to enable effective health promotion…[with] actions to improve health at three levels: the advancement of knowledge and skills among practitioners; the expansion of support and infrastructure for health promotion in organizations, and; the development of cohesiveness and partnerships for health in communities” [16]
Outcome Evaluation	An evaluation that measures changes the program has made in participants/clients based upon program objectives [17]
Learning Modality	Interventions, trainings, education, mentorships, delivered through face-to-face or in-person modes, online, or a combination of both [18].
Population Groups	Professional Personnel are individuals who received formal education and/or training in related global health fields (eg, doctors, nurses, professors, researchers etc…), Community Workers are individuals who have not received formal education and/or training (eg, community nurses, community health workers etc…), but who have some qualification in global health to practice within their community, and General Public (eg, community members, parents of school students etc…) includes individuals who do not have formal education and/or training, and who do not practice in any area related to global health [19]

LH conducted the search process, extracted all results into the Endnote software, and shared it with two reviewers (HN and MEH) who conducted the screening process after a calibration exercise. After excluding all duplicates, the two reviewers independently screened the titles and abstracts based on set criteria for eligibility. A third reviewer (MEK) was assigned to resolve disagreements. Next, full texts were retrieved, were scrutinized by the two initial reviewers, and conflicts were also resolved by the same third reviewer. Data were extracted into a pre-established Excel sheet in preparation for data synthesis and analysis, which was conducted on the Statistical Package for the Social Sciences (SPSS) (IBM, Armonk, NY, USA).

### Data analysis

The data was extracted by one reviewer (HN) and was analysed by two reviewers (HN, MEK). Besides the bibliography (which included the date, name of first author, title, region, and country) and summaries of included studies (which included duration of the initiatives, sample size, key findings, conclusions, learning outcomes, and limitations), two main categories of data were extracted and reported in **Table 2**. The first related to the GHCB initiative, and included data regarding the global health theme, design of the study, learning modality, sample size, and learning outcomes. The second related to the evaluation approach, and it included data regarding the level of evaluation, target population, method of evaluation, evaluation tool, administration frequency (time point of data collection), the measured outcome, and the conclusions and limitations.

**Table 2:** Summary of findings

Region	Topic	Design	Population	Modality	Level of Evaluation	Method of Evaluation	Time Point
**Africa**							
Asgary et al. (2016) [20]	NCD	Quantitative	Community Workers (Community health nurses)	Blended	Individual	Face-to-face	Post
Brantuo et al. (2014) [21]	SRH	Quantitative	Professional Personnel (health care providers; hospital nurses, doctors etc...)	Face-to-face	Individual & Organizational	Face-to-face & desk review	Pre-Post
Comeau et al. (2018) [22]	CD	Qualitative	Professional Personnel (Graduate students, postdoctoral trainees, academics etc...)	Blended	Individual	Face-to-face	Post
Crocker et al. (2016) [23]	Public health	Qualitative	Professional Personnel (Government officials)	Face-to-face	Individual & Organizational	Face-to-face	More than 3 mo
Davila et al. (2015) [24]	NCD	Mixed methods	Professional Personnel (epidemiologically trained hospital staff and ministry of health staff)	Face-to-face	Individual & Organizational	Face-to-face	More than 3 mo
Feldacker et al. (2014) [25]	Health system	Quantitative	Professional Personnel (Mid-level health care clinicians)	Face-to-face	Individual	Face-to-face	More than 3 mo
Garley et al. (2016) [26]	Health system	Quantitative	Professional Personnel (M&E Officers working in ministries of health, NGO, development projects etc…)	Blended	Individual	Face-to-face & online	More than 3 mo
Huber et al. (2014) [27]	Health system	Quantitative	Professional Personnel (Health practitioners, researchers, managers)	Face-to-face	Individual	Face-to-face	Post
Makanjuola et al. (2012) [28]	Mental health	Qualitative	Professional Personnel (mental health college teachers)	Face-to-face	Individual	Face-to-face	More than 3 mo
Matovu et al. (2013) [29]	Health system	Qualitative	Professional Personnel (Medical / nursing / clinical officers; social scientists etc…)	Face-to-face	Individual & Organizational	Face-to-face	Post
Mpofu et al. (2014) [30]	Health system	Mixed methods	Professional Personnel (health care students)	Face-to-face	Individual	Face-to-face	Post
Muchiri et al. (2016) [31]	NCD	Qualitative	General Public (adults diabetes patients)	Face-to-face	Individual	Face-to-face	More than 3 mo
Mutabaruka et al. (2010) [32]	Public health	Mixed methods	Professional Personnel (managers of immunisation programs)	Face-to-face	Individual & Organizational & Country	Face-to-face	Unspecified
O'Donovan et al. (2018) [33]	CD	Quantitative	Community workers (community health workers)	Blended	Individual	Face-to-face	Pre-Post
Plowright et al. (2018) [34]	SRH	Quantitative	Community workers (community health workers)	Face-to-face	Individual	Face-to-face	Pre-Post
Sarli et al. (2010) [35]	Health system	Qualitative	Community Workers (community health workers)	Face-to-face	Individual & Community	Face-to-face	More than 3 mo
Shimpuku et al. (2018) [36]	SRH	Mixed methods	General Public (pregnant women and family members)	Face-to-face	Individual	Face-to-face	Pre-Post
Sibeko et al. (2018) [37]	Mental health	Mixed methods	Community Workers (community health workers)	Face-to-face	Individual	Face-to-face	Pre-Post & up to 3 mo post
Tekola et al. (2019) [38]	Mental health	Qualitative	General Public (caregivers of children with developmental disorders) & Professional Personnel (Healthcare providers and other stakeholders)	Face-to-face	Individual	Face-to-face	Post
Wills et al. (2010) [39]	Health system	Qualitative	Community workers (Primary health care workers)	Face-to-face	Individual & Organizational	Face-to-face	Post
Witek-McManus et al. (2015) [40]	CD	Mixed methods	General Public (School teachers)	Face-to-face	Individual	Face-to-face	More than 3 mo
Fish et al. (2019) [41]	NCD	Mixed methods	Professional Personnel (health care providers; clinical and surgical oncologists)	Face-to-face	Individual	Online & Face-to-face	Pre-Post
**Asia:**
Abdel-All et al. (2018) [42]	NCD	Mixed methods	Community workers (accredited social health activists)	Face-to-face	Individual	Face-to-face	Pre-Post & up to 3 mo post
Carlos et al. (2015) [43]	CD	Quantitative	Professional Personnel (health care providers - doctors, nurses, medical technicians)	Face-to-face	Individual	Face-to-face	Pre-Post
Choi et al. (2016) [44]	Mental health	Quantitative	General Public (parents of adolescents)	Online	Individual	Online & Over the phone	Pre-Post & up to 3 mo post
Gao et al. (2018) [45]	Health system	Mixed methods	Professional Personnel (health workers, academics, researchers, government officials etc…)	Blended Learning	Individual	Online & Over the phone	Pre-Post & up to 3 mo post
Gyawali et al. (2018) [46]	NCD	Mixed methods	Professional Personnel (female community health volunteers)	Face-to-face	Individual	Face-to-face	Pre-Post
Jung et al. (2009) [47]	Health system	Mixed methods	General Public (community leaders)	Face-to-face	Individual	Face-to-face	Pre-Post
Kang et al. (2015) [48]	Health system	Quantitative	Professional Personnel (hospice and palliative health care experts)	Face-to-face	Individual	Face-to-face & Mailed survey	Post
Kusuma et al. (2019) [49]	CD	Quantitative	General Public (household samples)	Face-to-face	Community	Face-to-face	Pre-Post & up to 3 mo post
Limato et al. (2018) [50]	SRH	Qualitative	Community workers (community health workers) & General Public	Face-to-face	Individual & Community	Face-to-face	More than 3 mo
Monoto et al. (2018) [51]	SRH	Qualitative	Community Workers (Breastfeeding Peer Counsellors)	Blended Learning	Individual	Face-to-face	Pre-Post & up to 3 mo post
Negandhi et al. (2015) [52]	Health system	Mixed methods	Professional Personnel (Public health professionals)	Face-to-face	Individual	Online & Over the phone	Post
Nugroho et al. (2019) [53]	SRH	Quantitative	Community workers (outreach workers)	Face-to-face	Individual	Face-to-face	Pre-Post & up to 3 mo post
Shen et al. (2018) [54]	NCD	Quantitative	Community Workers (community nurses) & General Public (patients)	Face-to-face	Individual	Face-to-face	Pre-Post
Slakey et al. (2016) [55]	Surgical care	Qualitative	Professional Personnel (Healthcare providers)	Blended Learning	Individual	Online & Face-to-face	More than 3 mo
Sranacharoenpong et al. (2009) [56]	NCD	Quantitative	Community Workers (Community Healthcare Workers)	Blended Learning	Individual	Online & Face-to-face	Pre-Post
Wang et al. (2017) [57]	Mental health	Qualitative	Professional Personnel (Healthcare professionals - General practitioners and nurses	Face-to-face	Individual	Face-to-face	Pre-Post & up to 3 mo post
Xu et al. (2016) [58]	Health system	Quantitative	Professional Personnel (Undergraduate medical students)	Face-to-face	Individual	Face-to-face	Pre-Post
Yang et al. (2018) [59]	Mental health	Quantitative	Professional Personnel (Mental health professionals from colleges, hospitals, and health centers)	Face-to-face	Individual	Face-to-face	Pre-Post
Zhan et al. (2017) [60]	Health system	Quantitative	Community workers (Primary health care workers)	Blended Learning	Individual	Online & Face-to-face	Pre-Post
Zhang et al. (2012) [61]	Mental health	Quantitative	Professional Personnel (Physicians - pulmonogists)	Face-to-face	Individual	Face-to-face	Pre-Post
Zhao et al. (2019) [62]	SRH	Mixed methods	Professional Personnel (doctors, midwifes, directors, officers)	Face-to-face	Individual	Face-to-face	Post
Ziganshin et al. (2015) [63]	Health system	Quantitative	Professional Personnel (Physicians and medical trainees)	Face-to-face	Individual	Face-to-face	Post
**Caribbean:**							
Cianelli et al. (2013) [64]	Mental health	Qualitative	Professional Personnel (health care workers - nurses, physicians etc…)	Face-to-face	Individual	Face-to-face	Post
Knettel et al. (2017) [65]	SRH	Mixed methods	Community workers (Community health workers)	Face-to-face	Individual	Face-to-face	Pre-Post
Rivera et al. (2018) [66]	NCD	Quantitative	Professional Personnel (Public health graduate students)	Face-to-face	Individual	Face-to-face	Pre-Post
**Central America**							
Gonzalez et al. (2016) [67]	Public health	Quantitative	Professional Personnel (health care workers - physicians, nurses, nutritionists, respiratory technicians etc…)	Online	Individual	Online	Pre-Post
McConnell et al. (2017) [68]	Health system	Quantitative	Professional Personnel (nurses)	Online	Individual	Online	Pre-Post
Molokwu et al. (2016) [69]	NCD	Quantitative	General public (Primary care patients - women)	Face-to-face	Individual	Face-to-face	Pre-Post
**MENA:**
Abdelhai et al. (2012) [70]	SRH	Quantitative	Professional Personnel (Public Health Students)	Online	Individual	Online & Face-to-face	Pre-Post
Gholipour et al. (2018) [71]	Health system	Quantitative	Professional Personnel (heads of health centers, health managers, health deputies)	Face-to-face	Individual	Face-to-face	Pre-Post
Omar et al. (2009) [72]	Health system	Mixed methods	Professional Personnel (health managers)	Face-to-face	Individual & Organizational	Face-to-face	More than 3 mo
Siabani et al. (2016) [73]	NCD	Quantitative	General Public (patients with chronic heart failure)	Face-to-face	Individual	Face-to-face	Pre-Post & up to 3 mo post
**Pacific:**
Fung et al. (2015) [74]	Mental health	Qualitative	Professional Personnel (mental health clinicians)	Face-to-face	Individual	Online & Face-to-face	Pre-Post
Charlson et al. (2019) [75]	Mental health	Mixed methods	Professional Personnel (health care workers - nurses medical officers, counsellors)	Face-to-face	Individual	Face-to-face	Post
**International (multiple):**							
Khan et al. (2018) [76]	NCD	Quantitative	Professional Personnel (nurses, researchers, medical assistants etc…) & Community Workers (Community health workers)	Face-to-face	Individual	Face-to-face	Post
**South America**							
Gomes et al. (2015) [77]	NCD	Qualitative	Community Workers (community health workers)	Face-to-face	Individual	Face-to-face	More than 3 mo
Hull et al. (2012) [78]	Surgical care	Qualitative	Professional Personnel (post-graduate medical students)	Face-to-face	Individual	Face-to-face	Pre-Post
Joshi et al. (2011) [79]	Health system	Quantitative	Professional Personnel (health care providers - physicians, nurses, dentists) & Community workers (Community health workers)	Online	Individual	Online	Post
Monier et al. (2019) [80]	CD	Quantitative	Professional Personnel (post-graduate health care students)	Online	Individual	Online	Post
Pereira et al. (2015)[81]	Mental health	Quantitative	Professional Personnel (health care providers)	Blended Learning	Individual	Online	Pre-Post & up to 3 mo post
Vieira et al. (2014)[82]	Mental health	Quantitative	General Public (School teachers)	Face-to-face	Individual	Face-to-face	Pre-Post

CD – communicable disease, NCD – non-communicable diseases, SRH – sexual and reproductive health

Risk of bias and methodological quality of included studies was assessed using the McGill Mixed-Methods Appraisal Tool (MMAT) [83]. Since the aim of this review is to appraise evaluation approaches in GHCB, a meta-analysis was not applicable given that we did not examine effect sizes associated with the interventions. Instead, we opted for a qualitative and descriptive approach for data analysis.

## RESULTS

### Overview

**Figure 1:**
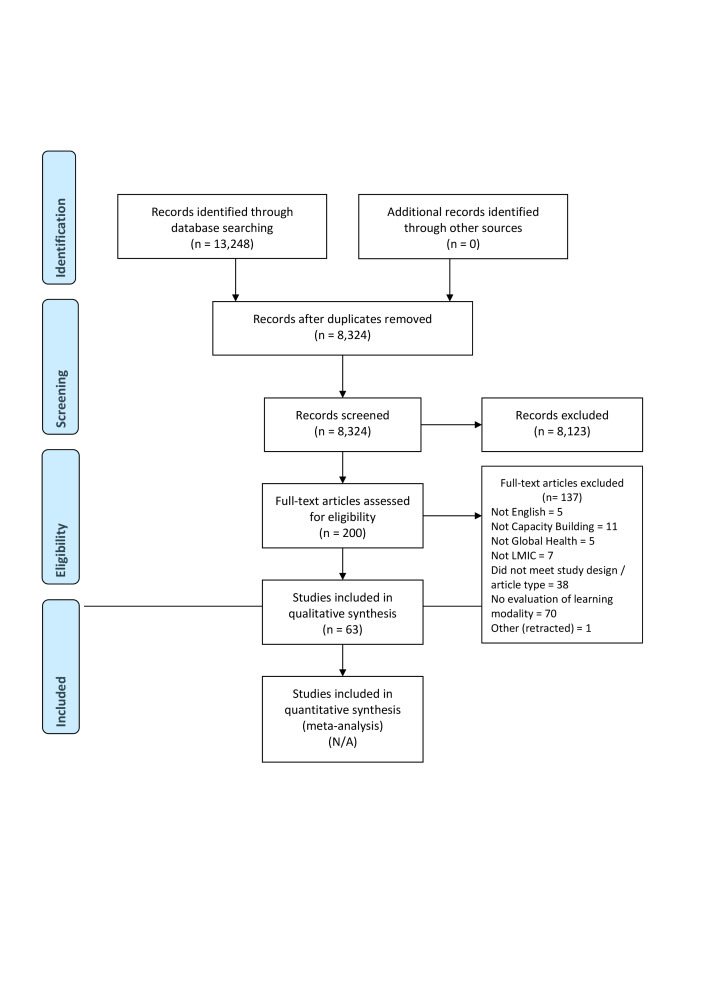
PRISMA flowchart.

Of the 8324 eligible articles, 200 studies met the inclusion criteria based on title and abstract screening and were eligible for full-text review, from which 63 articles were included for final analysis (**Figure 1**). Included studies (**Table 2**) reported initiatives conducted in Africa, Asia, Caribbean, Central America, Middle East and North Africa, Pacific, and South America. Global Health topics of the initiatives were categorized under mental health (19.0%), health system (28.6%), non-communicable diseases (20.6%), communicable diseases (9.5%) sexual and reproductive health (14.3%), public health (4.8%), and surgical care (3.2%).

### Characteristics of the studies

Three main population groups were addressed in the aforementioned initiatives, categorized into professional personnel (57.1%), community workers (20.6%), general public (14.3%) or mixed (7.9%). The most commonly adopted learning modality was face-to-face (74.6%), followed by blended (15.9%) and online learning (9.5%). Most evaluations were conducted on the individual level (84.1%), whereby only a handful of studies evaluated initiatives on the organizational, community, or country levels. The majority of studies relied on pre-and-post or only post intervention time intervals for evaluation (64.5%), and a small number reported up to 3 months follow-ups (16.1%) or more (19.4%). Capacity building initiatives were mostly evaluated using face-to-face methods (76.2%), and a limited number used online methods (7.9%) or a combination of online and other methods (15.9%). The most commonly used tools were questionnaires (63%), followed by open-ended or close-ended evaluation forms (49%), semi-structured interviews (35%), focus groups (25%), online surveys (22%), expert/peer rating (11%), case studies (9%), researcher observations (6%), desk reviews (3%), and reflective commentaries (2%).

Closer examination of the results revealed that African and Asian countries reported the most GHCB initiatives evaluations (69.8%). In comparison to other regions, they invested most in longer-term evaluations spanning 3 months or more (75%). African countries specifically, almost exclusively placed emphasis on evaluating modalities beyond the individual level. Across studies, GHCB initiatives were directed mostly towards professional personnel, and online evaluation methods were almost exclusively used for them. Online evaluation methods were only used for individual level evaluation, whereby other levels of evaluation such as organizational or community levels were only assessed face-to-face. Also, long-term evaluations spanning 3 months, or more, were conducted face-to-face. Results show that over the past 4 years, online methods of evaluation have been increasingly used in GHCB.

### Evaluation of learning modalities

With regard to learning modalities, blended and online GHCB initiatives were only evaluated on the individual level, whereby organizational, community, and country level evaluations were done only for face-to-face trainings. Similarly, long-term evaluations of 3 months or more were done for face-to-face modalities, whereby only 2 blended-approach initiatives - that had a face-to-face component - were evaluated at 3 months follow-up or more, and none for online approaches. Blended and online GHCB initiatives were also exclusively conducted for community workers and professional personnel, with the exception of 1 study that trained a sample of the general public through an online approach. Finally, results show that online and blended modalities are starting to increase in number over the past 4 years.

### Variables and indicators

In almost all capacity building initiatives, the main purpose was to improve the knowledge, capacity, skills, confidence, or performance of their participants. Evaluations and measured outcomes of the initiatives targeted (1) changes in knowledge, attitudes, skills, and performance, (2) perceptions, experiences, and satisfaction with the training, and (3) organizational, community, and country level impact in rare instances. Indicators for evaluations on the individual level focused on reactions, perceptions, satisfaction, experiences, and expectations, and on changes in knowledge, skills, practices, performance. Evaluations on the organizational level focused their indicators on assessing transfer (and barriers to application) of knowledge to the organization through learners’ technical input, performance, leadership, and relationship with colleagues, in addition to desk reviews of organizational-level changes such as policies, outputs, and practices. On the community-level, indicators mainly addressed the relationship between community members and health care services such as individuals’ knowledge of available services and related personnel, their health care practices, and their perceptions of health problems in the community.

### Study design

Articles used a mixture of qualitative (25.4%), quantitative (49.2%), and mixed-methods (25.4%) approaches, and all online studies were quantitative. Pre-and-post intervention changes along with satisfaction with trainings were generally measured quantitatively, whereas experiences and perceptions of the training were measured qualitatively. Organizational, community, and country level impact were assessed using one or both approaches. Finally, the GHCB initiatives overall improved knowledge, skills, and practices, whereby interactive modalities and engaging approaches were most important for knowledge retention and application. Online and blended modalities were reported to be generally effective and accepted by population groups, especially those that provide increased engagement and interaction. Common stated limitations included the need for longer-term and wider evaluations beyond individual-level learning and reaction.

### Risk assessment

Based on the MMAT tool used to evaluate the quality of studies in this review, a small number of studies (n = 9) scored low (2/4), and the rest (n = 54) scored higher (3/4 or 4/4) on quality assessment (**Table 3**).

**Table 3:** MMAT Risk of bias summary

Study ID (first author & year)	MMAT rating (out of ****)	Main limitations
**Abdel-All, 2018**	**	-Did not report response rate
		-Did not appropriately consider the limitations associated with integration of qualitative and quantitative data.
**Abdelhai, 2012**	***	-Unacceptable response rate
**Asgari, 2016**	***	-Measurements are not appropriate to answer the research question
**Brantuo, 2014**	**	-Measurements inappropriate.
		-Did not report the response rate.
**Carlos, 2015**	****	
**Charlson, 2019**	**	-Did not consider how findings relate to the context in which the data was collected
		-Did not consider how findings relate to the researchers influence.
**Choi, 2016**	***	-Unacceptable response rate
**Cianelli, 2013**	****	
**Comeau, 2018**	****	
**Crocker, 2016**	***	-Did not consider how findings relate to the researchers influence.
**Davila, 2015**	***	-Did not consider how findings relate to the researchers influence
**Feldacker, 2015**	****	
**Fish, 2019**	**	-Did not report the response rate
		-Did not take into account the difference between groups (did not list the demographic characteristics of the participant)
**Fung, 2015**	****	
**Gao, 2018**	****	
**Garley, 2016**	**	-Did not report the response rate
**Gholipour, 2018**	****	
**Gomes, 2015**	**	-Did not consider how findings relate to the researchers influence.
		-Did not consider how findings relate to the context in which the data was collected
**Gonzalez, 2016**	***	-Unacceptable response rate
**Gyawali, 2018**	***	-The integration of quantitative and qualitative data are not relevant to answer the research question
**Huber, 2014**	***	-Did not take into account the difference between groups (did not list the demographic characteristics of the participant)
**Hull, 2012**	****	
**Joshi, 2011**	**	Did not report the response rate
		-Did not take into account the difference between groups (did not list the demographic characteristics of the participant)
**Jung, 2009**	****	
**Kang, 2015**	****	
**Khan, 2018**	****	
**Knettel, 2017**	****	
**Kusuma, 2019**	****	
**Limato, 2018**	****	
**Makanjuola, 2012**	****	
**Matovu, 2013**	***	-Did not consider how findings relate to the researchers influence
**McConnell, 2017**	***	-Did not report response rate
**Molokwu, 2016**	***	-Unacceptable response rate
**Monier, 2019**	***	-Did not take into account the difference between groups (did not list the demographic characteristics of the participant)
**Monoto, 2018**	**	-Process for analyzing qualitative data are not relevant to answer the research question
**Mpofu, 2014**	***	-Did not report response rate
**Muchiri, 2016**	***	-Did not consider how findings relate to the researchers influence
**Mutabaruka, 2010**	***	-Unacceptable response rate
**Negandhi, 2015**	****	
**Nugroho, 2019**	****	
**O'Donovan, 2018**	****	
**Omar, 2009**	****	
**Pereira, 2015**	****	
**Plowright, 2018**	****	
**Rivera, 2018**	***	-Did not report the response rate
**Sarli, 2010**	****	
**Shen, 2018**	****	
**Shimpuku, 2018**	****	
**Siabani, 2015**	***	-Unacceptable response rate
**Sibeko, 2018**	****	
**Slakey, 2016**	***	-Did not consider how findings relate to the researchers influence
**Sranacharoenpong, 2009**	***	-Did not report the response rate
**Tekola, 2019**	***	Did not consider how findings relate to the researchers influence
**Vieira, 2014**	***	-Sample not representative of the population.
**Wang, 2017**	***	-Did not consider how findings relate to the researchers influence
**Wills, 2010**	***	-Did not consider how findings relate to the researchers influence
**Witek-McManus, 2015**	***	-The integration of quantitative and qualitative data are not relevant to answer the research question
**Xu, 2016**	**	-Did not report the response rate
		-Did not take into account the difference between groups (did not list the demographic characteristics of the participant)
**Yang, 2018**	***	-Did not report the response rate
**Zhan, 2017**	****	
**Zhang, 2012**	***	-Did not report response rate
**Zhao, 2019**	***	-Did not report response rate
**Ziganshin, 2015**	****	

MMAT – mixed-methods appraisal tool

## DISCUSSION

To the best of our knowledge, this is the first systematic review on the approaches used to evaluate GHCB initiatives in LMICs. To-date, only one review addressed capacity building tools [84], however it was limited to those targeting health research, a subcomponent of GHCB. Another systematic review addressed the effectiveness of capacity building initiatives in the public health domain [12], however authors did not focus on evaluation approaches. Both reviews, although did not focus on LMICs, highlighted the need for the development and refinement of more consistent evaluation approaches to measure larger and long-term impact of capacity building in general. Considering this increasing interest in capacity building, along with the noticeable knowledge gaps, the urgent need to cover a wider and more comprehensive scope of GHCB with a focus on evaluation approaches is well-warranted, especially in LMICs. This is a timely study given the advancements in capacity building delivery and evaluation methods, the growing global health burdens in LMICs, and the increasing need for the development of a competent health workforce in LMICs [1,85]. Evaluations are essential to assess the impact of GHCB initiatives on different levels, to assess whether or not their goals were achieved, and to highlight areas that may require improvements. Adequate evaluations are also important to gauge the utility of, and strategically allocate funds. We advance the literature by critically appraising the studies on this topic, by synthesising their findings, and by recommending increased standardization in evaluation approaches.

Overall, our findings indicate that most of the initiatives are delivered and evaluated through face-to-face modalities and this is not surprising, given the low resource settings that characterize LMICs, and their documented slow adoption of technology [86]. Interestingly however, based on our review, there has been a shift over the past 4 years towards integrating technology in the delivery and evaluation of GHCB initiatives. Of those, blended approaches that still include a face-to-face component are more prevalent than purely online modalities, potentially because such a transition is complex and may require more time, technological literacy, adequate infrastructure and cultural acceptance [86,87]. Nevertheless, such a shift may offer increased practicality and accessibility for the delivery and evaluation of GHCB initiatives given the potential of technology in overcoming barriers associated with traditional capacity building delivery methods [88]. For example, LMICs tend to have limited access to global health education, and technology has the potential to address this problem by allowing the transfer of knowledge from developed to developing countries in an easier, more accessible and cost-effective manner [88,89]. Our results indicate that clinically related GHCB initiatives included a face-to-face component, whereby purely online initiatives were didactic and did not include a hands-on approach. This may be an important avenue for future research, namely to assess the delivery of clinical practicums through online modalities. Finally, only one online intervention addressed the general public, whereby professional personnel and community workers were predominantly the target of the reviewed GHCB initiatives.

Our results build on previous research and point towards a growing need for better evaluation approaches for GHCB initiatives [12,84], especially for online and blended modalities. Customarily, higher-income countries tend to place more emphasis on adequate evaluation approaches to assess higher level and longer-term impact of their initiatives on the individual, organizational, and community levels in comparison to LMICs [84]. In one systematic review, most of the included studies that assessed long-term organizational-level impact of capacity building were from higher-income countries [84]. Evidently, higher levels of evaluation are time consuming, and require more sophisticated and complex methods of measurement [90]. In this review, while a small number of face-to-face approaches evaluated their initiatives long-term, and assessed impact beyond individual level, none of the online initiatives did so, and only 2 blended GHCB initiatives conducted evaluations beyond the 3 months-time period. As a result, we were not able to draw further conclusions regarding blended and online modalities beyond the individual-level assessment of reaction, knowledge, attitudes, and practices, or their long-term efficacy. This is especially important since blended and online approaches were effective and accepted by the participants, which means that a shift toward technology-assisted GHCB may be promising [67,88]. There is thus an urgent need to refine evaluation approaches used in initiatives delivered through technology-assisted modalities in order to better assess their wider and longer-term impact. While individual-level assessment offers important first-level insight, it does not tap into higher levels of assessing organizational or community-level changes such as shifts in policy, practices, or access to health care services to name a few. One way to do that is through the standardization of evaluation approaches, a process by which assessments are developed to have uniform applications with consistent procedures across studies [91].

Standardization may simplify and provide a better structure for the evaluation of initiatives based on the GHCB modality, vis-à-vis population groups, evaluation methods, level of evaluation, frequency of evaluation, and evaluation tools, and may enhance comparability of outcomes. That said, we identified much variability in the evaluation methods and tools used across studies, whereby evaluations deviated from standardization in terms of the lack of consistency in the items/questions, set variables, indicators, and outcomes. Also, few studies reported the actual tools that were used, and this is problematic because it dissipates the efforts and resources allocated towards the evaluation of GHCB, in that authors tended to develop their own tools rather than use or adapt existing ones. It is important for future studies to be more consistent in their evaluation approaches. Ultimately, this means that GHCB initiatives in LMICs need standardized evaluation tools to measure the effectiveness of learning modalities beyond the well-addressed individual-level assessment of knowledge, attitude, and practice. These tools may be adapted to the context and approach of each initiative.

Results of this study should be interpreted in light of several limitations. Most significantly, the reviewed articles are not representative of GHCB initiatives among LMICs, given that we only included those evaluating the outcome of at least one component of the modality. Also, studies may have been missed due to the search parameters used, which is a common problem in the literature. However, we minimized this by exhausting the search process through the key terms, and search strategy employed across multiple academic and grey literature databases. In addition, many LMICs may prioritize reporting to donors as opposed to publishing their findings, which may affect the accurate representation of studies included in this review. Finally, we had a limited focus on demographic groups and other related capacity building data, because it was beyond the scope of the review: our main goal was to review the methods and evaluation approaches.

## CONCLUSION

This review provides the first summary of evaluation approaches used in GHCB in LMICs and identifies several gaps that need to be addressed. Most significantly, because online and blended modalities are promising avenues for GHCB, more attention should be placed on evaluating them long-term beyond the individual level in order to maximize their impact and sustainability. In addition to that, standardized evaluation tools for GHCB are needed in order to enhance efficiency and comparability of results across initiatives.

## Additional material

Online Supplementary Document
